# Supplementary material for: Discovery and application of insertion-deletion (INDEL) polymorphisms for QTL mapping of early life-history traits in Atlantic salmon
Source: BMC Genomics. 2010 Mar 8;11:156. doi: 10.1186/1471-2164-11-156 (PMC2838853; doi:10.1186/1471-2164-11-156)
Supplement: Additional file 2 — Information on developed 76 locus single-run INDEL panel in Atlantic salmon. Information on fluorescence labeling, primer concentrations, PCR pooling and links to alignments, INDEL motifs and GENESCAN (Burge and Karlin 1997) predictions of genes/exons are available in html format. [file 1471-2164-11-156-S2.ZIP › Additionalfile2/snpsummary20056.html]

```
Cluster 10615 Contig 1

prev  Summary    Contig List  next
```

Size of Consensus sequence = 833

Number of sequences = 4

Minimum redundancy = 1

Key

A gi|89853421|gb|DY709544.1|DY709544 EST\_ssal\_rgb2\_65283 ssalrgb2 mixed\_tissue Salmo salar cDNA Salmo salar cDNA clone ssal\_rgb2\_605\_191\_rev 5', mRNA sequence  
B gi|117297887|gb|EG647700.1|EG647700 SGP258876 Atlantic salmon normalized Brain cDNA library Salmo salar cDNA clone NB1-0985 5', mRNA sequence  
C gi|89852515|gb|DY708638.1|DY708638 EST\_ssal\_rgb2\_64377 ssalrgb2 mixed\_tissue Salmo salar cDNA Salmo salar cDNA clone ssal\_rgb2\_603\_346\_rev 5', mRNA sequence  
D gi|85019162|gb|DW547818.1|DW547818 EST\_ssal\_rgb2\_12237 rgb2 Salmo salar cDNA clone ssal\_rgb2\_520\_241\_rev 5', mRNA sequence

5 SNPs detected

A B C D  cosegregation weighted

192 . G - -   2/5 30.00
569 . - - C   3/5 45.00
570 . - - A   3/5 45.00
571 . - - G   3/5 45.00
580 . A G G   2/5 30.00
